# Correspondence

**Published:** 1969-07

**Authors:** P. M. Bennett

**Affiliations:** Ham Green Hospital, Pill


CORRESPONDENCE

Dear Sir,

I was very interested in "The Management of Spina Bifida" by Mr. J. B. M. Roberts, F.R.C.S., speaking as a ward sister caring for this type of child. From experience I find that the majority of parents are most confused, as they are passed from one doctor to another, each giving various opinions, which makes it very difficult for them to understand the child.

When the children are admitted for assessment, one hears what little knowledge the parents have; and this is why I think a combined clinic is important, as only a small one is held at Southmead Hospital. Cardiff seems to have control of this problem, and I hope that Bristol will quickly follow.

Yours, etc.,

P. M. Bennett,
(Sister)

